# Comparison of AOD from CALIPSO, MODIS, and Sun Photometer under Different Conditions over Central China

**DOI:** 10.1038/s41598-018-28417-7

**Published:** 2018-07-03

**Authors:** Boming Liu, Yingying Ma, Wei Gong, Ming Zhang, Wei Wang, Yifan Shi

**Affiliations:** 1 State Key Laboratory of Information Engineering in Surveying, Mapping and Remote Sensing (LIESMARS), Wuhan University, Wuhan, China; 2 Collaborative Innovation Center for Geospatial Technology, Wuhan, 430079 China

## Abstract

Cloud-Aerosol Lidar and Infrared Pathfinder Satellite Observation (CALIPSO) provides three-dimensional information on aerosol optical properties across the globe. However, the performance of CALIPSO aerosol optical depth (AOD) products under different air quality conditions remains unclear. In this research, three years of CALIPSO level 2 AOD data (November 2013 to December 2017) were employed to compare with the Moderate Resolution Imaging Spectroradiometer (MODIS) level 2 columnar AOD products and ground-based sun photometer measurements for the same time period. To investigate the effect of air quality on AODs retrieved from CALIPSO, the AODs obtained from CALIPSO, MODIS, and sun photometer were inter-compared under different air quality conditions over Wuhan and Dengfeng. The average absolute bias of AOD between CALIPSO and sun photometer was 0.22 ± 0.21, 0.11 ± 0.07, and 0.14 ± 0.13 under clean, moderate, and polluted weather, respectively. The result indicates that the CALIPSO AOD were more reliable under moderate and polluted days. Moreover, the deviation of AOD between CALIPSO and sun photometer was largest (0.23 ± 0.21) in the autumn season, and lowest (0.13 ± 0.12) in the winter season. The results show that CALIPSO AOD products were more applicable to regions and seasons with high aerosol concentrations.

## Introduction

Aerosols have a significant influence on global climate change by directly altering solar radiation or indirectly modifying the cloud properties^1–3^. However, estimation of aerosols’ radiative force is uncertain due to a lack of knowledge of height-revolved optical properties^4–7^ and the nonhomogeneous spatial distribution of aerosol particles^8^. A variety of satellite sensors, such as Moderate Resolution Imaging Spectroradiometer (MODIS), Multi-angle Imaging Spectroradiometer (MISR), and Visible Infrared Imaging Radiometer Suite (VIIRS), have been used for long-term continuous detection of aerosol optical depth (AOD)^9–11^. However, due to the limitations of passive satellite measurement, they can only provide the total column value and not the vertical distribution information for aerosols, which is important for assessment of aerosol radiative effects^12^. The Cloud-Aerosol Lidar and Infrared Pathfinder Satellite Observation (CALIPSO) was developed to provide atmospheric vertical profile detecting capabilities to fill the current observation gap^13^.

The CALIPSO instrument can detect the atmospheric vertical profile at one depolarization and two scattering channels^14^. Information on the vertical distribution of clouds and aerosols is necessary to improve the estimation accuracy of direct and indirect radiative forcing by aerosols, and to assess the feedback of clouds within the climate system^15^. Currently there are many studies comparing CALIPSO vertically integrated extinction with AODs from other satellite sensors (MODIS and MISR), as well as the ground-based Aerosol Robotic Network (AERONET)^16,17^. Kittaka *et al*. assessed the consistency of AODs obtained from the CALIPSO aerosol layer product version 2 and the MODIS-Aqua collection 5 from June 2006 to August 2008. They indicated that the two sensors have good correlation over ocean regions with low cloudiness^18^. Schuster *et al*. compared CALIPSO column AODs with ground-based AERONET sites from June 2006 to May 2009, and found that the CALIPSO dust retrievals had a larger assumed LiDAR ratio (more than 40 sr)^19^. Yu *et al*. compared AODs from CALIPSO, Goddard Chemistry Aerosol Radiation and Transport (GOCART) model simulations, and MODIS observations from June 2006 to November 2007, and suggested that MODIS AODs were generally larger than CALIPSO observations over most regions^20^. Liu *et al*. evaluated the performance of CALIPSO AOD products on the high-value regions and low-value regions, and studied the seasonal variation of AOD products over China^21^. Such comparisons provide valuable insights into the application of CALIPSO products.

With the increase in anthropogenic emissions and the advancement of urbanization, dust and haze pollution occur frequently over central China^22^. More and more studies have used the AOD products on the environmental research^3,5,11^. Wang and Ma *et al*. employed the satellite AOD data to derive the PM_2.5_^23,24^. These studies all require a good accuracy of AOD data. But some studies have indicated that the degree of air pollution would affect satellite AOD inversion^25^. Li *et al*. compared MODIS and AERONET AODs over China, and indicated that AODs retrieved from MODIS have larger errors in extreme aerosol conditions^26^. Chen *et al*. evaluated the performance of the MODIS AOD product under haze-fog pollution conditions over Beijing, and pointed out that the accuracy and spatial coverage of the MODIS 3 km Dark Target AOD is poor on heavily polluted days^27^. Therefore, it is important to assess the bias of AOD products under the different pollution level. However, there are still few studies evaluating CALIPSO AODs under different air quality conditions. The effect of air quality on CALIPSO AOD inversion needs further research, which is important for the application of CALIPSO AODs in regional environmental research.

In this work, CALIPSO AOD product was assessed by validating against the 550-nm column AOD obtained from the MODIS products and ground-based sun photometer measurements obtained at two ground sites over central China. The performance of CALIPSO AOD product was evaluated on the different PM2.5 concentrations. When taking into consideration the effect of air quality on CALIPSO AOD inversion, we find that concentrations of fine particulate matter (PM2.5) play an important role in the consistency of comparisons. Moreover, differences in AOD between CALIPSO and sun photometer measurements have obvious seasonal characteristics.

## Results

### Comparison of AODs from CALIPSO, MODIS, and Sun Photometer

**Figure 1:**
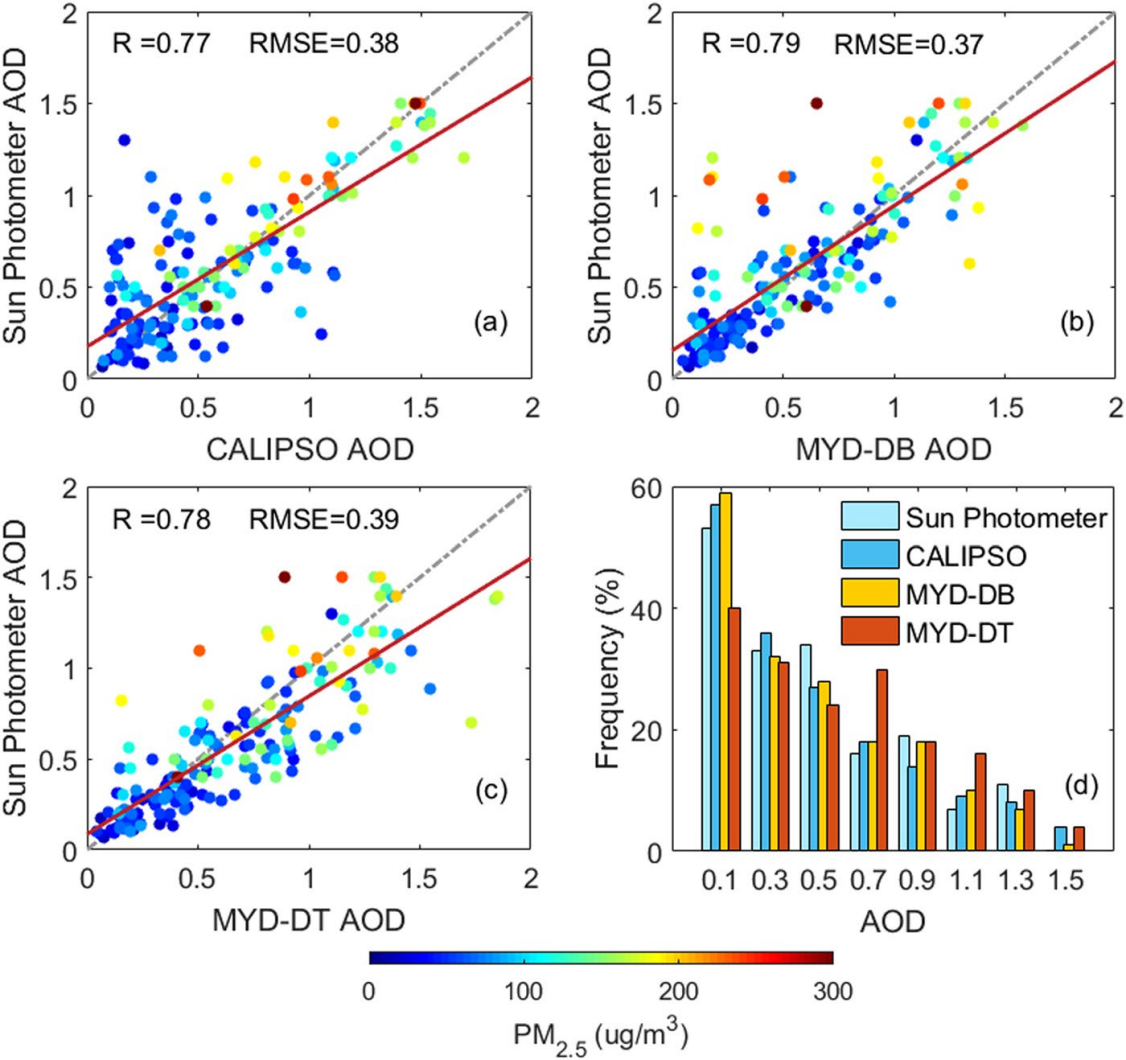
Scatter plots of the AODs derived from CALIPSO, MODIS, and sun photometer with change of PM2.5 from November 2013 to December 2017. (**a**) CALIPSO vs. sun photometer; (**b**) MODIS-DB vs. sun photometer; (**c**) MODIS-DT vs. sun photometer; (**d**) frequency distribution of AODs. Color bars represent the mass concentration of PM2.5.

Figure 1 shows scatter plots of the AODs derived from CALIPSO, MODIS, and sun photometer with change of PM2.5. Color bars represent the mass concentration of PM2.5. Figure 1a shows a comparison of AODs obtained from CALIPSO and sun photometer. They exhibit good correlation with each other; R = 0.77 and RMSE = 0.38. Figure 1b shows a comparison of AODs retrieved from MYD_DB and sun photometer, with R = 0.79 and RMSE = 0.37. Figure 1c shows a comparison of the AODs derived from MYD_DT and sun photometer, with R = 0.78 and RMSE = 0.39. This indicates that the AODs obtained from CALIPSO and MODIS have good correlation with the ground-based observations. Other evaluation studies have shown similar results^18,19^. Moreover, the color bar suggested that AODs are more similar when the mass concentration of PM2.5 is low. Figure 1d shows the frequency distribution of AODs retrieved from CALIPSO, MODIS, and sun photometer. The results show that their frequency distributions are similar.

### Comparison of AODs under Different Air Quality Conditions

To investigate the effect of air quality on AODs retrieved from CALIPSO, the AODs obtained from CALIPSO, MODIS, and sun photometer were compared under different air quality conditions. The experimental data were divided into three subsets, based on the latest air quality standards of China (GB 3095-2012)^28^. A 24 h average PM2.5 > 150.0 µg m^−3^ indicates heavy air pollution, 24 h average PM2.5 > 75.0 and < 150 µg m^−3^ indicates moderate air pollution, and 24 h average PM2.5 < 75 µg m^−3^ indicates clean air quality. According to the air quality standards of China, the experimental data were classified as 110 days of clean weather, 48 days of moderate air pollution, and 30 days of heavy air pollution.

**Figure 2:**
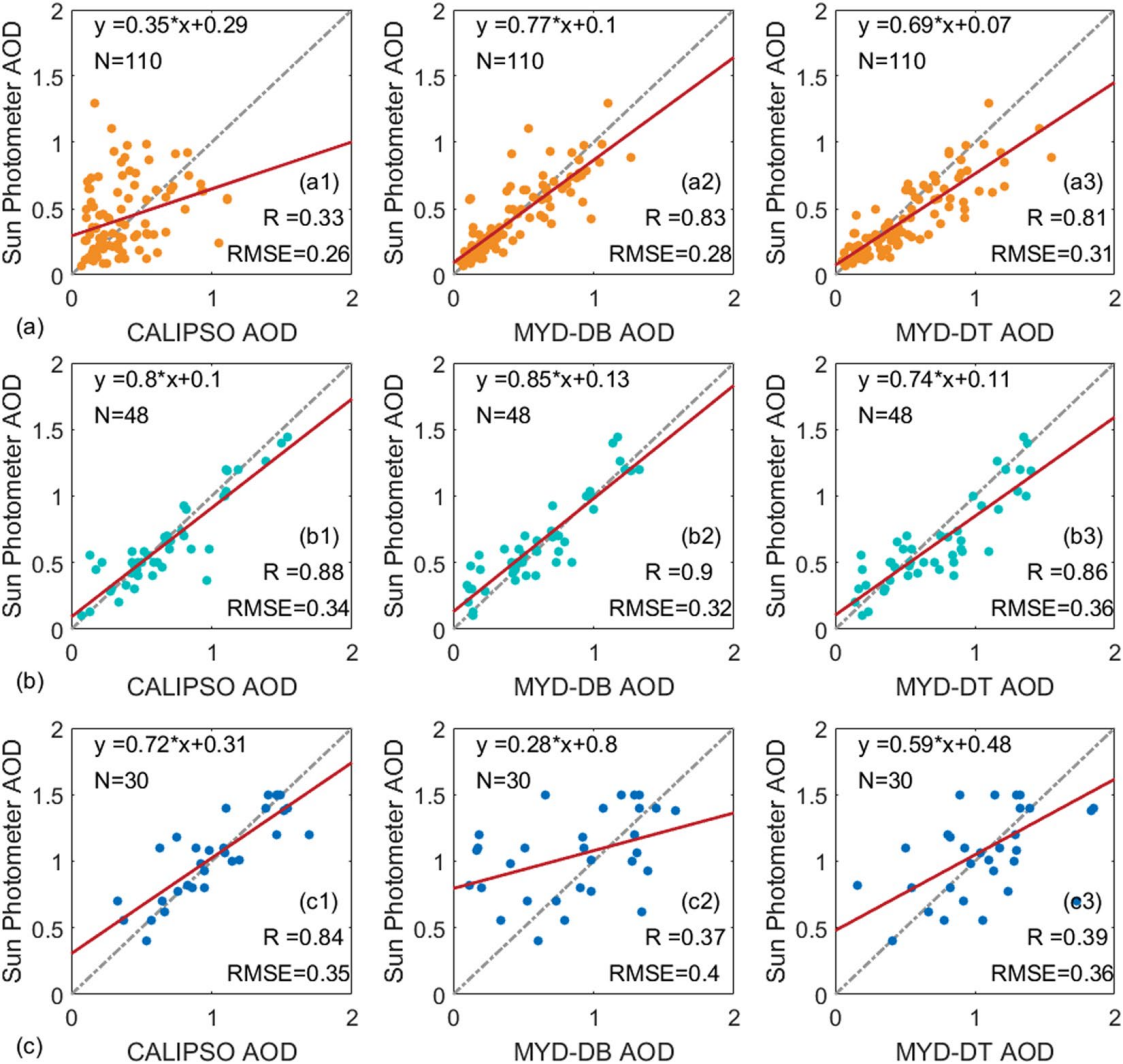
Scatter plots of AODs derived from CALIPSO, MODIS, and sun photometer under different air quality conditions. (**a**) Orange, (**b**) green, and (**c**) blue points represent sample points under clean weather, moderate air pollution, and heavy air pollution, respectively. Red and grey lines represent the linear regression curve and 1:1 reference curve, respectively.

Figure 2 shows comparisons of AODs derived from CALIPSO, MODIS, and sun photometer under different air quality conditions. Figure 2a shows a comparison of AODs derived from CALIPSO, MODIS, and sun photometer under clean weather. The correlation coefficients between CALIPSO and sun photometer, MYD_DB and sun photometer, and MYD_DT and sun photometer were R = 0.33, R = 0.83, and R = 0.81, respectively. Compared with the ground-based sun photometer, AODs retrieved from CALIPSO showed lower correlation than MODIS under clean weather. During moderate air pollution (Fig. 2b), the correlation coefficients between CALIPSO and sun photometer (R = 0.88) show obvious improvement. The correlation coefficient between MYD_DB (DT) and sun photometer reached R = 0.9 (0.86). This suggests that AODs derived from CALIPSO, MODIS, and sun photometer have good consistency under moderate air pollution. However, Fig. 2c shows that the correlation between CALIPSO and sun photometer AOD (R = 0.84) was better than that between MYD_DB/DT and sun photometer (R = 0.37/0.39) under heavy air pollution. This indicates that CALIPSO AODs exhibit poor correlation with sun photometer under clean weather. Meanwhile, the AODs retrieved from MODIS showed poor performance under dust events or heavy urban/industrial haze. This result was similar to previously validated research, where the MODIS product exhibited poor performance under extreme aerosol conditions over East Asia^17,20,26^.

**Figure 3:**
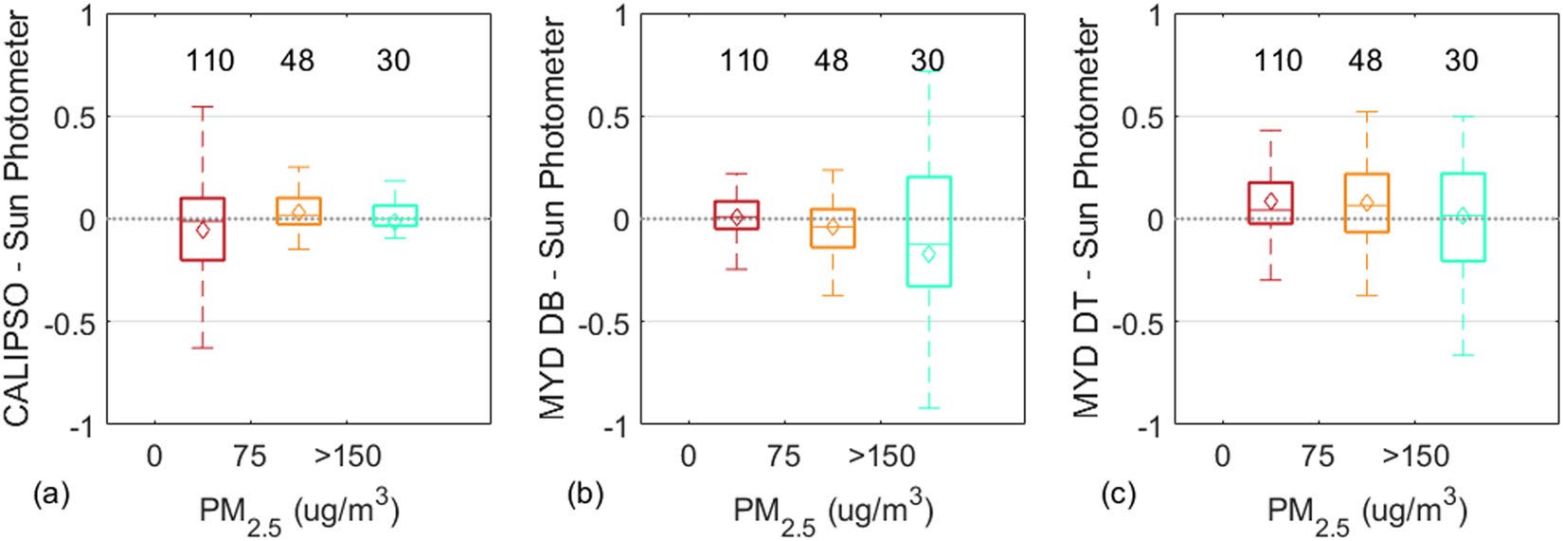
Box plots of AOD differences between (**a**) CALIPSO and sun photometer; (**b**) MODIS-DB and sun photometer; and (**c**) MODIS-DT and sun photometer with PM2.5 concentration change over Central China. The numbers represent the sample size of each box. The middle line and square show the median and mean value of the AOD differences, respectively.

Figure 3 shows box plots of AOD differences between CALIPSO and sun photometer, MYD_DB and sun photometer, and MYD_DT and sun photometer with PM2.5 concentration change over central China. Figure 3a shows that the AOD difference between CALIPSO and sun photometer was small when the PM2.5 concentrations was high. The AOD difference between CALIPSO and sun photometer was largest (RMSE = 0.31) in the range of 0 to 75 ug/m^3^ PM2.5 concentrations. Figure 3b shows the AOD difference between MYD_DB and sun photometer with a PM2.5 concentration change. This indicates that the AOD difference between MODIS and sun photometer was increased with increased PM2.5 concentrations. The larger AOD difference (RMSE = 0.45) between MYD_DB and sun photometer was in the range of PM2.5 >150 ug/m^3^. Figure 3c shows the AOD difference between MYD_DT and sun photometer with the PM2.5 concentration change. It shows that the smallest AOD difference (RMSE = 0.3) between MYD_DT and sun photometer was in the range of PM2.5 <75 ug/m^3^. These results show that air quality can affect satellite AOD retrieval due to the limitations of the AOD inversion method^11,27^. The reasons are described in the Discussion section.

### Seasonal deviation of AODs under Different Air Quality Conditions

**Figure 4:**
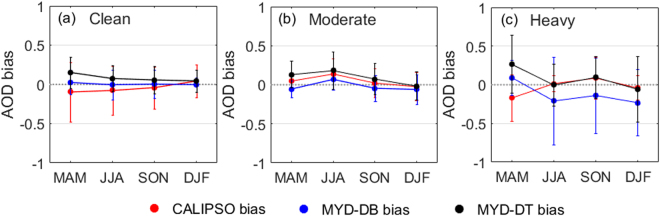
The seasonal deviation of AOD products under the different air quality conditions (**a**) The clean air quality (PM2.5 < 75 µg m–3); (**b**) the moderate air pollution (75 < PM2.5 < 150 µg m–3); and (**c**) the heavy air pollution (PM2.5 > 150.0 µg m–3). The red, blue, and black lines show the CALIPSO bias, MYD_DB bias, and MYD_DT bias, respectively.

Figure 4 shows the seasonal deviation of CALIPSO, MYD_DB, and sun photometer AOD products under the different air quality conditions. Figure 4a shows that the seasonal deviation of AOD products under clean air quality. The results indicated that the seasonal variation of AOD deviations are the same under clean air quality, the deviation of AOD was smallest in winter season and largest in spring season. Figure 4b shows that the seasonal deviation of AOD products under moderate air pollution. The seasonal variation of AOD deviations is also consistent. Figure 4c shows that the seasonal deviation of AOD products under heavy air pollution. The seasonal variation of MYD_DB AOD deviations are similar with MYD_DT AOD deviations, but different with CALIPSO AOD deviations. The result shows that the deviation of CALIPSO AOD was smallest under summer (0.01 ± 0.1) and winter (−0.04 ± 0.15) season. It indicated that CALIPSO AODs were more suitable for the summer and winter season under heavy air pollution.

### Regional deviation of AODs

**Figure 5:**
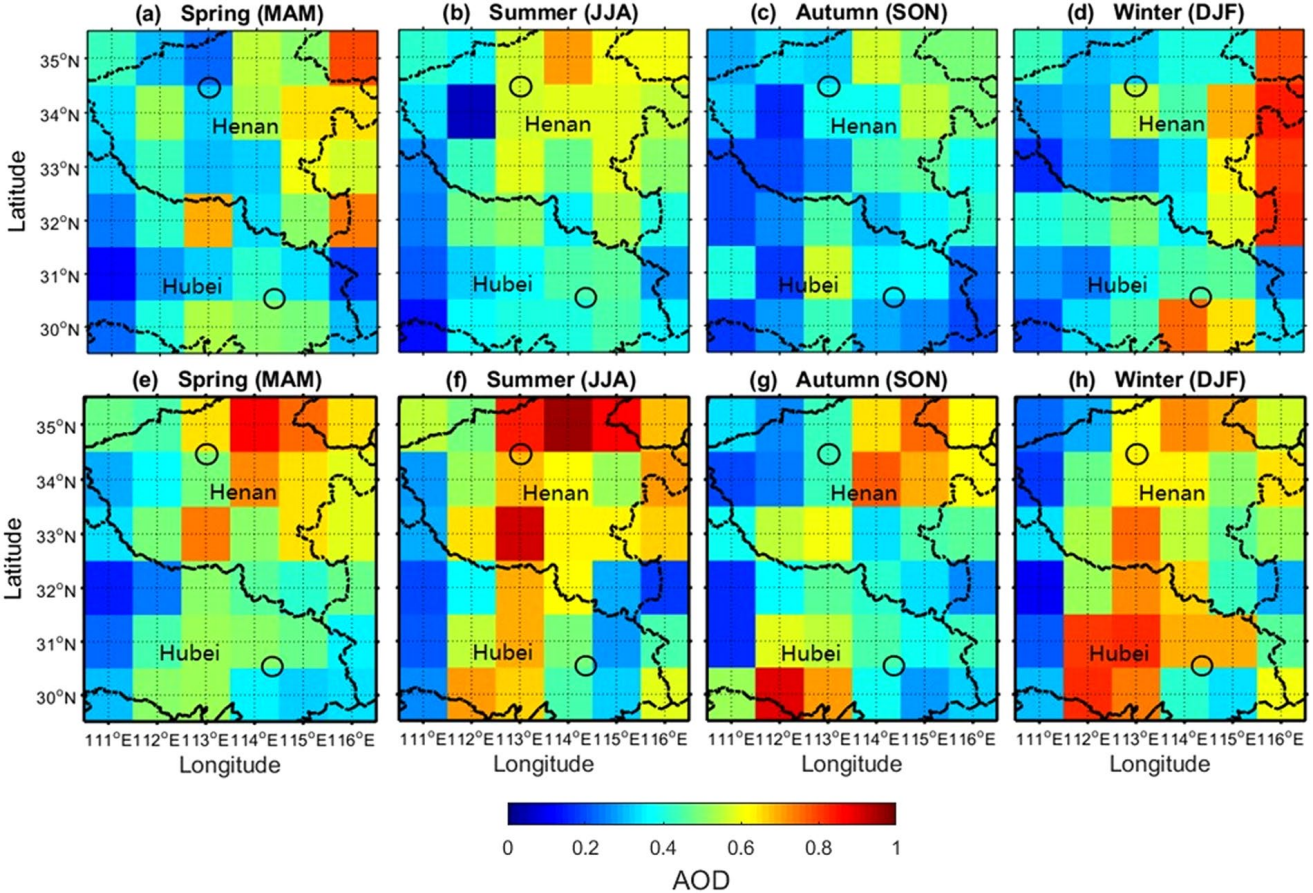
Seasonal mean distributions of (**a**–**d**) CALIPSO AODs and (**e**–**h**) MYD_DB AODs from November 2013 to December 2017 on 1° × 1° equal-angle grids. Black circle represents the ground-based observation station. Spring: March, April, May; summer: June, July, August; autumn: September, October, November; winter: December, January, February.

**Figure 6:**
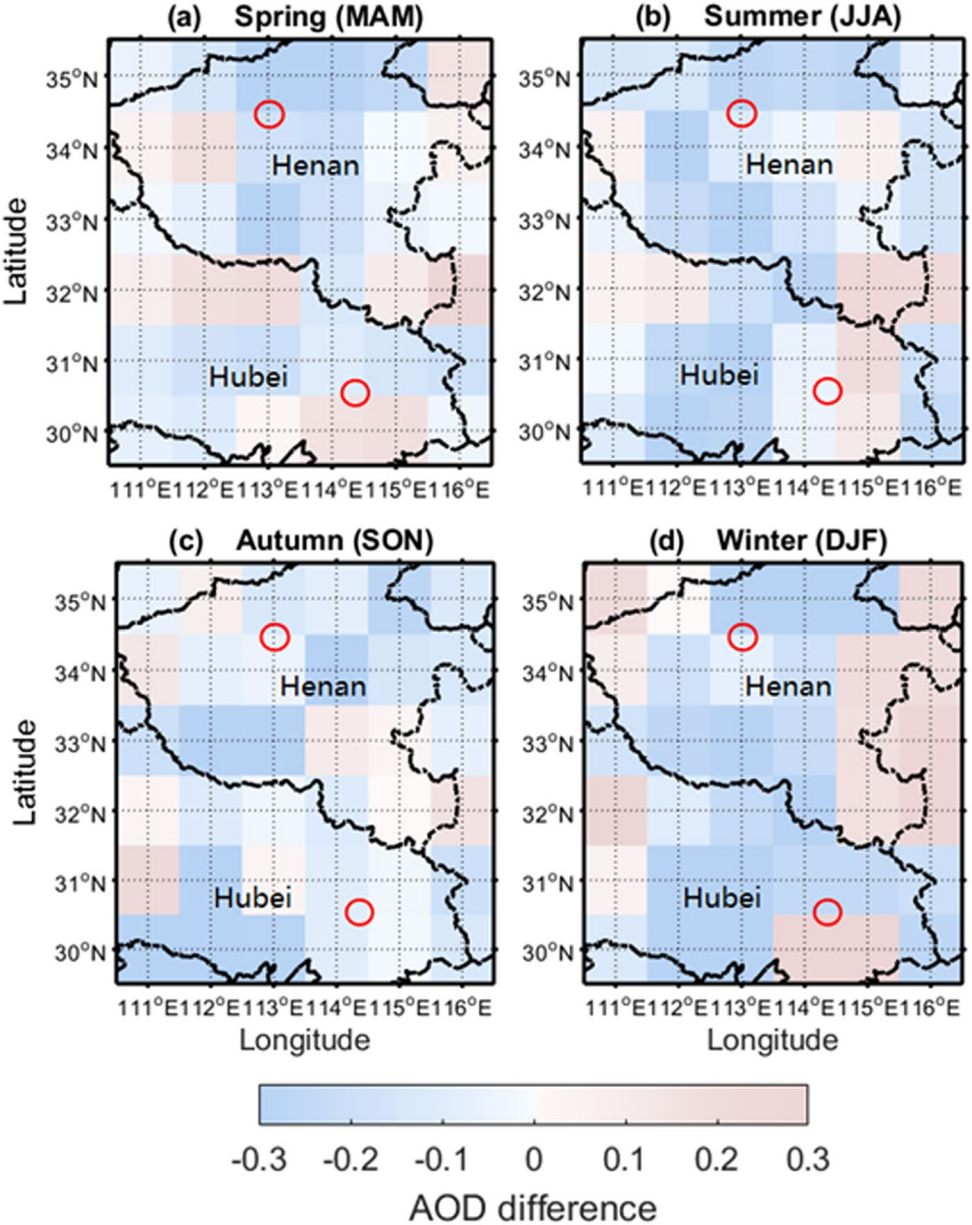
Seasonal mean differences of MYD_DB and CALIPSO AODs from November 2013 to December 2017 on 1° × 1° equal-angle grids. Red circle represents the ground-based observation station. (**a**) Spring: March, April, May; (**b**) summer: June, July, August; (**c**) autumn: September, October, November; (**d**) winter: December, January, February.

Figure 5 shows the seasonal mean distributions of CALIPSO AODs and MODIS AODs from November 2013 to December 2017 on 1° × 1° equal-angle grids. The spatial distribution exhibited by CALIPSO was consistent with MODIS, e.g., the maximum AOD occurs over northeast Henan due to agricultural fires and anthropogenic emissions^29^. The seasonal average of CALIPSO AOD over central China is respectively 0.438, 0.429, 0.344 and 0.396 from spring seasons to winter seasons. The mean MODIS AOD over central China is 0.529, 0.542, 0.456 and 0.482, respectively, for spring, summer, autumn and winter seasons. It is found that mean AOD over central China is high in spring and summer, and relatively low in autumn and winter. The larger AOD load during spring and summer can be attributed to the high relative humidity and anthropogenic emissions. The increasing industrial and other human activities emitted the pollutant particles. Moreover, the high relative humidity during summer may lead to hygroscopic growth of aerosol particles, resulting in larger AOD. The relatively low AOD values during spring and autumn were due to the Mongolian monsoon^22^. Atmospheric conditions with low pressure and high wind speed effectively promote circulation of the atmosphere, leading to a low aerosol load^30^. However, there were some differences between CALIPSO and MODIS AODs, shown in Fig. 6.

Figure 6 shows seasonal mean differences of MODIS and CALIPSO AODs from November 2013 to December 2017 on 1° × 1° equal-angle grids. The differences shown in Fig. 6 were defined as CALIPSO_AOD–MODIS_AOD, which describes the AOD difference between CALIPSO and MODIS retrievals. Meanwhile, the significance test (σ = 0.05) with regard to the AOD differences were conducted. The test statistics of four seasons are 6.23, 6.51, 9.43 and 3.99, respectively. All test statistics are greater than the critical value (3.98). It indicated that the AOD difference is significant. Overall, most CALIPSO AODs were systematically lower than those obtained from MODIS over Central China, especially in the Southeast Hubei and Mount Song observation station areas. Over high-pollution areas, such as East Henan, the differences between MODIS and CALIPSO AODs have obvious seasonal features. CALIPSO AODs are larger than MODIS AODs during spring and winter, and smaller in summer and autumn.

## Discussion

As outlined above, the AODs retrieved from CALIPSO were assessed through a comparison with MODIS and sun photometer AODs. Overall, CALIPSO AODs are well correlated with sun photometer AODs, and can provide a solid dataset for quantitative research and air quality monitoring over central China. But AODs obtained from CALIPSO appear to have large error (>0.2) on very clean days.

**Figure 7:**
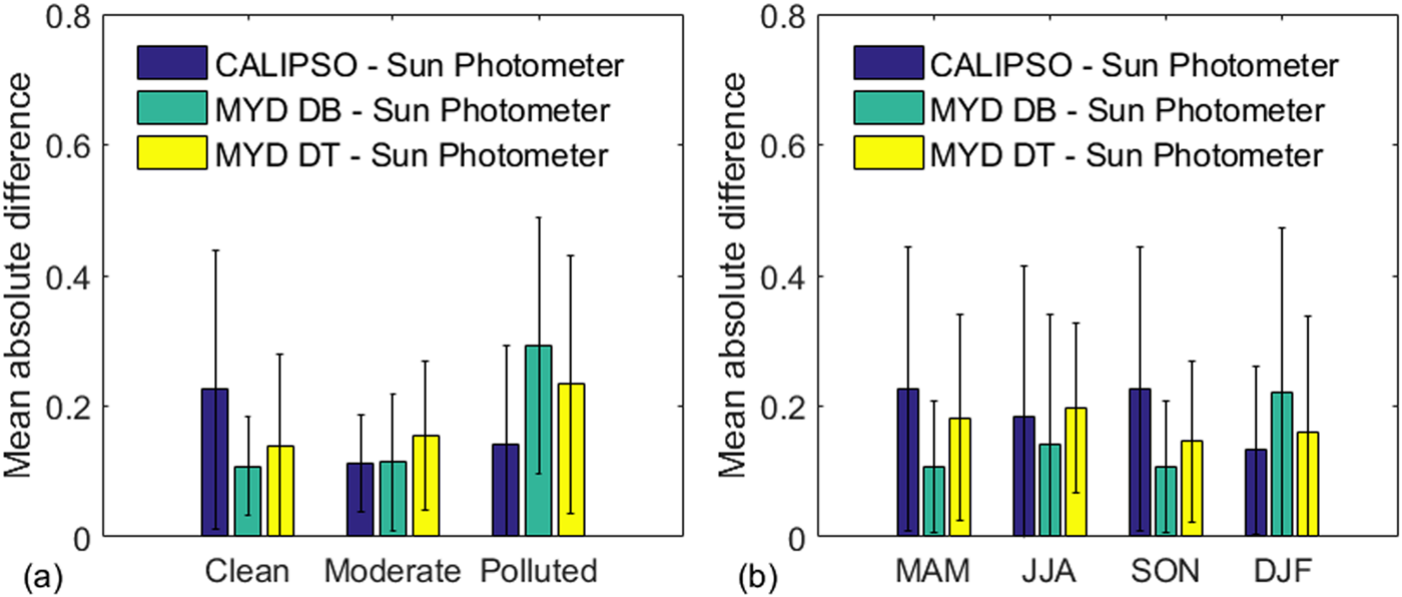
Average absolute bias of AODs under different conditions: (**a**) clean, moderate, and polluted conditions; and (**b**) in March, April May; June, July, and August; September, October, and November; and December, January, and February. Error bars represent standard deviations.

Figure 7a shows the average absolute bias of AOD under different air quality conditions. Average absolute bias was relatively low on moderate pollution days. The average absolute bias of AOD between CALIPSO and sun photometer, MYD_DB and sun photometer, and MYD_DT and sun photometer was 0.11 ± 0.07, 0.11 ± 0.1, and 0.16 ± 0.15, respectively. Based on ground-based observation data, the largest average absolute bias of AOD between CALIPSO and sun photometer was 0.22 ± 0.21 under clean weather. The largest average absolute bias of AOD between MYD_BD and sun photometer was 0.29 ± 0.19 under polluted conditions. The results in Figs 2 and 7a confirm that air quality does affect satellite AOD inversion. CALIPSO AODs exhibit poor correlation with sun photometer under clean weather. The AODs retrieved from MODIS showed poor performance under heavy air pollution. These differences were probably due to different AOD inversion methods^8,17^. It should be noted that CALIPSO retrieves AOD based on the aerosol extinction vertical profile, which is classified as different types of aerosol layers by the scene classification algorithm^31,32^. But the signal-to-noise ratio under clean weather is often too low to accurately search the weak aerosol layers on the aerosol extinction vertical profile^8,19^. This means that highly diffuse and/or tenuous scattering aerosol layers that lie below the CALIPSO detection threshold would be ignored by CALIPSO estimates of column AOD. As a consequence, weak aerosol layers that are not detected would not be retrieved, which would result in decreased retrieved AODs under clean weather. This is also the reason that AODs obtained from CALIPSO are generally lower than those from MODIS (Fig. 6). As for the inversion of MODIS AODs, surface reflectance, single scattering albedo, and aerosol model assumptions were important input parameters in the MODIS AOD inversion algorithm^26,27^. However, it was difficult to obtain accurate surface reflectance and single scattering albedo under heavy air pollution. Heavy industrial haze and highly concentrated secondary aerosols may have affected the input parameters in the MODIS AOD inversion algorithm^26^. Thus, AODs retrieved from MODIS exhibited poor performance under extreme aerosol conditions. These results indicate that the CALIPSO AODs had high accuracy for moderate and polluted days over Central China.

Figure 7b shows the average absolute bias of AODs in different seasons. The largest average absolute bias of AOD between CALIPSO and sun photometer was 0.23 ± 0.21 in the autumn season, and the lowest average absolute bias was 0.13 ± 0.12 in the winter season. Due to the Mongolian monsoon during autumn^22,30^, atmospheric conditions with low pressure and high wind speed effectively promoted circulation of the atmosphere, leading to the low aerosol load. The CALIPSO AOD over Central China was lowest (0.344) in autumn. The low AOD load may result in the larger error on AODs retrieved from CALIPSO in autumn. The relatively low average absolute bias of AOD between CALIPSO and sun photometer during the winter season can be attributed to frequent haze pollution, resulting in a high aerosol load over central China. Previous studies have indicated that the haze pollution occurred frequently under summer and winter season^22,29^. The high aerosol load was favourable to the CALIPSO AOD inversion, but it was adverse to the MODIS AOD inversion. It was also the reason that the average absolute bias of AOD between MYD_DB and sun photometer was the largest (0.22 ± 0.25) during the winter season. The results show that CALIPSO AODs were more reliable during summer and winter than spring and autumn over central China.

## Conclusions

In this study, three years of CALIPSO level 2 AOD data were employed to compare the MODIS level 2 columnar AOD products and ground-based sun photometer measurements over the same time period from November 2013 to December 2017. The results show that the AODs obtained from CALIPSO and sun photometer exhibit good correlation with each other, with R = 0.77 and RMSE = 0.38. Moreover, the air quality does affect satellite AOD inversion. The CALIPSO AOD have a larger uncertainty in clean days. The largest average absolute bias of AOD between CALIPSO and sun photometer was 0.22 ± 0.21 under clean weather. It was due to the limitation of satellite AOD inversion method. CALIPSO retrieved the AOD by the method of aerosol layer integral, which was easy to miss the thin layer, leading to the larger deviation. By contrast, the CALIPSO AOD were relatively reliable under the moderate (0.11 ± 0.07) and polluted (0.14 ± 0.13) days over central China. The enhanced aerosol layers can be effectively detected by CALIPSO, so the deviation of CALIPSO AOD was reduced with increased PM2.5 concentrations. Lastly, the seasonal analyses show that largest average absolute bias of AOD between CALIPSO and sun photometer was 0.23 ± 0.21 in the autumn season, and the lowest average absolute bias was 0.13 ± 0.12 in the winter season. This indicates that CALIPSO AODs were more suitable for the summer and winter season, when the haze pollution occurred frequently. On the contrary, the largest average absolute bias of AOD between MYD_BD and sun photometer was 0.29 ± 0.19 under polluted conditions. The performance of MODIS AOD was poor over heavy haze and only proposed valid data when the air is clean. In summary, When the aerosol load was relatively high over Central China, the CALIPSO AOD can provide a solid dataset for air quality monitoring. However, AODs retrieved from CALIPSO under the clean days were vulnerable to the tenuous scattering aerosol layers, and improvements and modifications are needed to achieve good accuracy.

## Materials and Data

### Observation Area

With rapid economic growth and population expansion, central China suffers from serious environmental and pollution problems, which have significantly increased AOD^31,33^. To obtain enough study cases, two ground-based observation stations were used to collect the sun photometer data: Wuhan and Mountain Song. The Wuhan observation station is located at the State Key Laboratory of Information Engineering in Surveying, Mapping and Remote Sensing (LIESMARS), Wuhan University (30°32′N, 114°21′E). The sun photometer was installed on the LIESMARS roof ^34^. The Mountain Song observation station is located in Dengfeng City in Henan Province (34°31′N, 113°07′E). The sun photometer was on the roof of a building at the Songshan observation station^30^. The hourly mean PM2.5 mass concentrations were obtained from the Ministry of Environmental Protection of the People’s Republic of China’s observation network (http://datacenter.mep.gov.cn/).

### AOD from Sun Photometer

The fully automated CE-318 sun photometer is a high-precision solar and sky radiation measuring instrument for use in environmental, meteorological, marine, and remote sensing applications^35^. The instrument is mainly used to measure solar and sky radiation in visible and near-infrared wavelengths to calculate the physical and optical properties of atmospheric aerosol, as well as characteristics of atmospheric optical thickness, turbidity, water vapor, ozone, etc. Detailed instrument calibration and AOD inversion methods are described in^36^. The uncertainty of AOD is approximately 0.01 to 0.02^37^. Note that the sun photometer could not directly inverse the AOD at 532 nm wavelength, which matched the CALIPSO visible wavelength, so we interpolated between available sun photometer wavelengths based on the spectrum Angstrom index relationship to get the AOD at the required wavelength^38^.

### AOD from MODIS

The MODIS is an important sensor mounted on the Terra and Aqua satellites, which are morning and afternoon satellites, respectively. MODIS satellite data includes information about ground surface albedo, cloud boundaries, atmospheric water vapor, aerosol, surface temperature, and other characteristics^39^. Satellite coverage in the Central China region cycles twice daily, which means that it can provide data about Central China twice a day. In this study, the MYD_DB and MYD_DT AOD at 550 nm from the MODIS-Aqua Level 2 aerosol data product were obtained, and only the highest-quality-flag (QF = 3) AOD observations were considered for analysis. It should be explained that the wavelengths of MODIS and CALIPSO AOD inversion are different, 550 nm and 532 nm, respectively. However, this difference is around 2–4%, much smaller than the differences between AOD inversion with different instruments^40^. Thus, differences caused by different wavelengths can be neglected in this study.

### AOD from CALIPSO

The CALIPSO satellite provides global observational data on aerosol and cloud layers to study the effect of clouds and aerosols on Earth’s climate^41^. The cycle time of this satellite across the central China region is 16 days. The CALIPSO satellite can detect vertical aerosol extinction profiles and aerosol subtypes including smoke, dust, polluted dust (dust and smoke), and clean and polluted continental and clean marine subtypes^32^. We used the CALIPSO 5 km Aerosol Layer Product (Level 2, Version 3), which provides aerosol layer optical depth at a wavelength of 532 nm. Moreover, the cases with cloud layer were removed in our comparisons. CALIPSO retrieves AOD based on the scene classification algorithm and the integrated aerosol extinction vertical profile^42^.

## Comparison of Methods

According to the statistical methods of other studies^9,10^, the root-mean-square error (RMSE; Equation (1)), the mean absolute difference (MAD; Equation (2)), and the correlation coefficient (R) were applied to evaluate the uncertainty in aerosol algorithms. The equation of linear regression was used to calculate the slope and intercept of the sample data.

1 $$RMSE=\sqrt{\frac{1}{n}\sum _{i=1}^{n}{(AO{D}_{(satellite)i}-AO{D}_{(ground)i})}^{2}}$$

2 $$MAD=\frac{1}{n}\sum _{i=1}^{n}|AO{D}_{(satellite)i}-AO{D}_{(ground)i}|$$

The experimental data were collected from November 2013 to December 2017. Based on previous data-matching principles^10^, the satellite AOD products were useful when at least 20% of the pixels fell within the ground observation station. Meanwhile, the satellite data compared with sun photometer measurements were the average of satellite AOD products within a range of 20 km at the ground observation station. The ground-based sun photometer data were obtained within 30 min of CALIPSO overpass times. The CALIPSO satellite crosses the ground-based observation station every 16 days. To ensure time synchronization, the MODIS and sun photometer data were screened based on the CALIPSO date. Note that the sun photometer data obtained from Wuhan and Mount Song stations were regarded as ground-based observation data over central China. After the screening process, there were 188 valid matchups cases: 96 and 92 satellite passes within 20 km over the Wuhan and Mount Song stations, respectively.
